# Membrane-anchored calpains – hidden regulators of growth and development beyond plants?

**DOI:** 10.3389/fpls.2023.1289785

**Published:** 2023-12-19

**Authors:** Martin Šafranek, Alain Shumbusho, Wenche Johansen, Júlia Šarkanová, Stanislav Voško, Boris Bokor, Ján Jásik, Viktor Demko

**Affiliations:** ^1^Institute of Botany, Plant Science and Biodiversity Centre, Slovak Academy of Sciences, Bratislava, Slovakia; ^2^Department of Plant Physiology, Faculty of Natural Sciences, Comenius University in Bratislava, Bratislava, Slovakia; ^3^Faculty of Applied Ecology, Agricultural Sciences and Biotechnology, Inland Norway University of Applied Sciences, Hamar, Norway; ^4^Comenius University Science Park, Comenius University in Bratislava, Bratislava, Slovakia

**Keywords:** calpain proteases, cell fate, development, DEFECTIVE KERNEL1 (DEK1), membrane-anchored calpains, nonclassical calpains

## Abstract

Calpains are modulatory proteases that modify diverse cellular substrates and play essential roles in eukaryots. The best studied are animal cytosolic calpains. Here, we focus on enigmatic membrane-anchored calpains, their structural and functional features as well as phylogenetic distribution. Based on domain composition, we identified four types of membrane-anchored calpains. Type 1 and 2 show broad phylogenetic distribution among unicellular protists and streptophytes suggesting their ancient evolutionary origin. Type 3 and 4 diversified early and are present in brown algae and oomycetes. The plant DEK1 protein is the only representative of membrane-anchored calpains that has been functionally studied. Here, we present up to date knowledge about its structural features, putative regulation, posttranslational modifications, and biological role. Finally, we discuss potential model organisms and available tools for functional studies of membrane-anchored calpains with yet unknown biological role. Mechanistic understanding of membrane-anchored calpains may provide important insights into fundamental principles of cell polarization, cell fate control, and morphogenesis beyond plants.

## 1 Introduction

Calpains (Clan CA, family C02; EC 3.4.22.17) are cysteine proteases that modify diverse cellular substrates by limited proteolysis (Ono and Sorimachi, 2012). Cleavage by calpain can lead to substrate’s activation or de-activation, intracellular relocation, or destabilization and subsequent degradation (Jeong et al., 2011; Ono & Sorimachi, 2012; Piatkov et al., 2014a). Calpains play critical roles in wide range of cellular processes including cell cycle regulation (Jánossy et al., 2004), cell motility (Franco and Huttenlocher, 2005; te Boekhorst et al., 2022) and cell fate determination (Santos et al., 2012; Kim et al., 2019). Their loss of function cause embryonic lethality in animals and plants (Takano et al., 2011; Olsen et al., 2015). Majority of what we know about calpain ‘s mode of action is based on “classical” members of the superfamily. Besides their physiological functions, calpain activities have been implicated in number of human diseases and pathologies, including limb-girdle muscular dystrophy, neurodegenerative disorders, or tumour pathogenesis(Huang and Wang, 2001; Storr et al., 2015; Ahmad et al., 2018). Pathological functions of calpains have been associated with their insufficient activity or hyperactivity, leading to suboptimal or excessive cleavage of their substrates, respectively. Mechanistic understanding of calpains regulation and their downstream targets processing is therefore instrumental for the development of tools interfering with specific disease-associated calpain activities (Sorimachi et al., 2011; Ono and Sorimachi, 2012).

The majority of calpain proteases are defined as cytosolic, not anchored to biological membranes via transmembrane domain (Sorimachi et al., 2011). In fact, all vertebrate and insect calpains are cytosolic (Araujo et al., 2018). Nevertheless, membrane association seems to play important role in the activation of cytosolic calpains (Araujo et al., 2018). For instance, activation of human CAPN2 by epidermal growth factor-mediated association with membrane phosphatidylinositol 4,5-bisphosphate was reported (Shao et al., 2006; Leloup et al., 2010). Although not containing a transmembrane domain, the CALPA in Drosophila contains a cluster of 16 hydrophobic amino acids with possible role of anchoring the protease to a cell membrane. Jékely and Friedrich (1999) proposed a model where CALPA is activated by phosphatidylinositol phosphates and released from cell membrane upon autolysis. Besides cytosol, intra-organellar localization of calpains has been also reported in number of cases, such as nuclear and mitochondrial localization of mammalian CAPN1 and CAPN2 (Tremper-Wells and Vallano, 2005; Kar et al., 2010; Chukai et al., 2021).

Membrane-anchored calpains with unique domain composition have been identified in plants, and similar sequences have been found in diverse microorganisms (Lid et al., 2002; Ono and Sorimachi, 2012). They belong to non-classical calpains and represent one of the four ancestral calpains with origin dating more than billion years ago (Liang et al., 2013). Despite potentially essential roles of membrane-anchored calpains, our knowledge about their 3D structure and biological function remains largely unknown.

## 2 Calpain´s mode of action as learned from classical cytosolic members of the superfamily

Based on the structure and domain composition, calpains are classified as classical, and non-classical, and all share a cysteine protease core domain (CysPc) harboring the catalytic triad of cysteine (Cys), histidine (His) and asparagine (Asn) residues (Strobl et al., 2000; Moldoveanu et al., 2002; Hanna et al., 2008; Sorimachi et al., 2011). In humans for example, there are 15 genes encoding calpain proteases, nine of them classified as classical. The classical calpains typically consist of an N-terminal helix, a CysPc, a calpain-type beta-sandwich domain (CBSW, formerly called C2-domain-like, C2L) and a penta -EF-hand (PEF) domain. Ubiquitously expressed classical calpains, such as human CAPN1 and CAPN2 form heterodimers with calpain small subunits CAPNS1 and CAPNS2, respectively (Hosfield, 1999; Strobl et al., 2000). Heterodimerization has also been described between the tissue-specific CAPN8 and CAPN9 (Hata et al., 2016). Zhao et al. (2012) reported 41 calpain domain architectures in prokaryotes and eukaryotes, mostly belonging to non-classical calpains (Zhao et al., 2012). The non-classical calpains typically do not form heterodimers with small subunits and differ in domain composition, mainly lacking the N-terminal helix and PEF domain while containing additional domains such as microtubule interacting and transport motif, a zinc finger containing domain or a C2 domain (Dear et al., 1997; Matena et al., 1998). Some non-classical calpains (e.g., mammalian CAPN6) lack one or more conserved active site residues in their CysPc domain and have non-proteolytic functions (Tonami et al., 2013; Hata et al., 2016).

Structural studies of classical calpains revealed an intriguing mechanism of the CysPc activation involving a calcium-mediated electrostatic switch triggering conformational changes within the CysPc (Suzuki et al., 1981; Elce et al., 1997). Calcium (Ca^2+^) binding triggers specific movements of flexible peptide loops leading to the rotation of the CysPc subdomains PC1 and PC2 relative to each other and the realignment of the active sites. The peptide loops within PC1 and PC2 are mechanistically essential and contribute to diverse requirements for calcium between different calpain types. For instance, a recently solved 3D structure of human non-classical CAPN5 revealed unique structural features of its CysPc domain with flexible loops playing an important role in disease-associated protease activity (Bondada et al., 2021). Classical calpains are inhibited by endogenous calpain inhibitor calpastatin, a protein encoded by the *CAST* gene (Moldoveanu et al., 2004; Sato et al., 2011; Yu et al., 2020).

Several prediction programs for preferential calpain cleavage sites have been developed (Shinkai-Ouchi et al., 2016a) it is generally accepted that diverse factors contribute to calpain’s cleavage site recognition, such as higher order of protein structure rather than primary amino acid sequence (Tompa et al., 2004; Shinkai-Ouchi et al., 2016b; Liu et al., 2019). Significant substrate specificity differences across individual calpains have also been reported (duVerle and Mamitsuka, 2019). Therefore, the identification of calpain *in vivo* substrates is still challenging and relies on applied experimental approaches. Diverse substrates have been reported in mammalian cells. They include receptor kinases, membrane transporters, phosphatases, phospholipases, cytoskeletal proteins, transcription factors, calmodulin-binding proteins, cell cycle regulators, and many others (Chan and Mattson, 1999; Piatkov et al., 2014b). Examples of disease-associated substrates include huntingtin (Huntington disease, (Gafni and Ellerby, 2002), parkin (Parkinson’s disease, (Shams et al., 2019; Gao et al., 2022), sodium calcium exchangers, the CDK5 activators p35 and p25, dynamin-like protein 1 (Alzheimer’s disease), (Ferreira, 2012; Kurbatskaya et al., 2016; Mahaman et al., 2019), or sarcomere proteins (Jia et al., 2001; Vainzof, 2003).

## 3 The only membrane-anchored calpain with known biological role is plant DEFECTIVE KERNEL1

Plants colonized land around 515–494 million years ago (Harris et al., 2022). Land plants evolved from charophyte algae that diverged from chlorophyte lineages around 1 billion years ago (Dagan et al., 2013; de Vries et al., 2016). Although chlorophyte algae possess only cytosolic calpains, most recent charophytes retained both cytosolic and membrane-anchored calpains (Zhao et al., 2012). However, land plants possess a single calpain, the membrane-anchored DEFECTIVE KERNEL 1 (DEK1) (Lid et al., 2002; Zhao et al., 2012; Liang et al., 2013; Perroud et al., 2014). It has been proposed that cytosolic calpains were lost during the transition from charophytes to land plants, and DEK1 provided an important function in increasing the morphological complexity of evolving plant bodies (Demko et al., 2014; Perroud et al., 2014).

Initially, the *DEK1* gene was identified in maize mutants lacking nutrient-rich aleurone cells in their seeds (Lid et al., 2002).Aleurone cells form a cytologically and functionally highly specialized layer around the starchy endosperm. The aleurone cell fate depends on their surface position relative to starchy endosperm and DEK1 function (Gruis et al., 2006). Induced loss of DEK1 function in maize endosperm resulted in trans-differentiation of aleurone cells into starchy endosperm cells, and reversibly, after the gene restoration, conversion of surface-positioned starchy endosperm cells to aleurone cells was observed (Yi et al., 2011a). Later it has been shown that without DEK1, plants are unable to form any organized tissues, and the protein plays essential roles in cell division plane orientation and cell fate maintenance beyond endosperm, starting from early embryogenesis and throughout post-embryonic development (Johnson et al., 2005; Lid et al., 2005; Perroud et al., 2014; Olsen et al., 2015). All this has prompted genetic studies of DEK1 in diverse plant models including maize, Arabidopsis, tobacco, rice, and the moss *Physcomitrium patens* (Lid et al., 2002; C. Wang et al., 2003; Ahn et al., 2004; Tian et al., 2007a; Johnson et al., 2008; Hibara et al., 2009; Demko et al., 2014; Perroud et al., 2014; Amanda et al., 2017).

**Figure 1:**
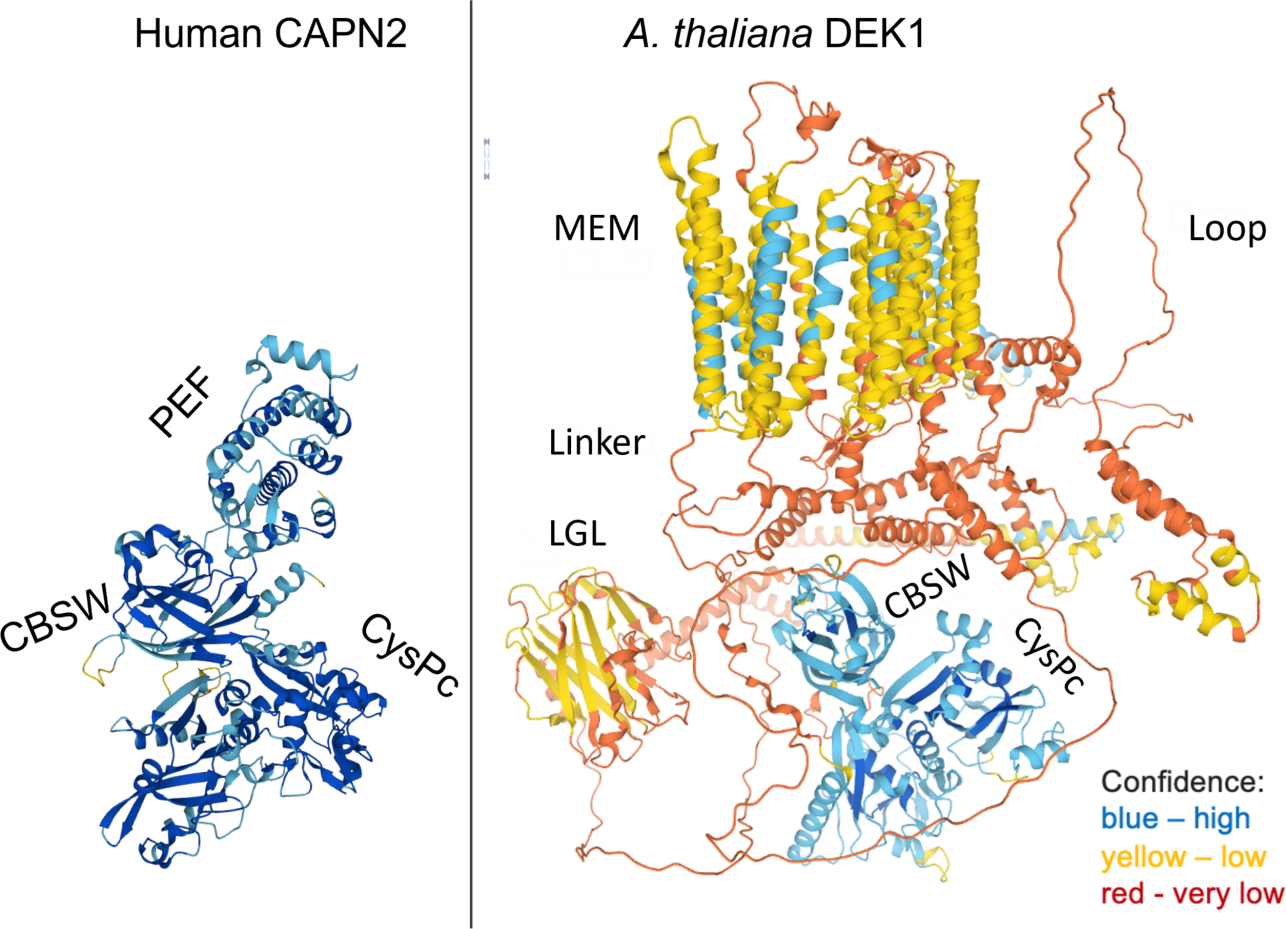
The structures of human CAPN2 as a representative of cytosolic classical calpain and *A. thaliana* DEK1 (AtDEK1) as a member of membrane-anchored non-classical calpain. Note that CAPN2 and DEK1 share only a calpain moiety composed of a protease core domain CysPc and a CBSW domain. The 3D models were retrieved from the AlphaFold Protein Structure Database (Varadi et al., 2022).

Based on structure prediction, DEK1 is a 240 kDa multi-domain protein composed of a 23-spanning transmembrane domain (MEM) with a long central Loop, a Linker with a Laminin_G-like domain (LGL) and a C-terminal calpain protease composed of a catalytic core CysPc and a calpain-type beta-sandwich domain (CBSW) (C. Wang et al., 2003; Johansen et al., 2016)(**Figure 1**). DEK1 is highly conserved in land plants with more than 80% amino acid identity of its CysPc domain between mosses and angiosperms (Liang et al., 2013).

Several conserved domains have been experimentally analyzed in DEK1. The most conserved part of DEK1 across land plants is its C-terminal calpain. Loss-of-function mutations that prevent translation of the DEK1 calpain cause embryonic lethality in angiosperms and developmental arrest of leafy shoots (gametophores) in the moss *P. patens* (Perroud et al., 2014). When expressed under the ubiquitous pRPS5A promoter, the DEK1 calpain moiety itself (CysPc-CBSW) is capable to complement the embryo lethal *dek1-3* mutation in the model plant *Arabidopsis thaliana* (Johnson et al., 2008; Liang et al., 2013). Similarly, reintroducing the *P. patens* DEK1 (PpDEK1) calpain part into the *DEK1* deletion mutant has rescued arrested gametophore development in this moss (Perroud et al., 2014). The calpain from PpDEK1 is also capable to rescue *A. thaliana dek1-3* mutant (Lid et al., 2005; Hibara et al., 2009; Liang et al., 2013). As common for all proteolytically active calpains in eukaryotes, the active site of DEK1 CysPc is formed by the Cys, His and Asp residues (Berti & Storer, 1995; Hosfield, 1999; Wang et al., 2003). Point mutation of the active site Cys (Cys-to-Ser substitution, *calp^null^*) abolished maize DEK1 calpain activity *in vitro* (Wang et al., 2003) and the AtDEK1 *calp^null^* variant failed to complement the *dek1-3* mutant in *A. thaliana* (Liang et al., 2013). In *P. patens*, the calpain active site Cys mutation in the full DEK1 protein (*dek1^null^*) phenocopies the entire *PpDEK1* gene deletion, indicating that calpain is an effector of a whole protein (Johansen et al., 2016). The DEK1 CysPc also contains two conserved Ca^2+^ binding sites with essential functions, as demonstrated by site-directed mutagenesis in *A. thaliana* (Liang et al., 2013). Experimental manipulation of *DEK1* transcript levels in *A. thaliana* and *P. patens* pointed to the association of the calpain function with cell wall remodeling and cell fate control (Liang et al., 2013; Amanda et al., 2016).

Altogether, the genetic analyses have shown that the calpain protease of DEK1 is essential for plant development. How exactly the multi-spanning transmembrane domain and Linker domain contribute to the DEK1 function, and especifically to the calpain regulation, remains largely unknown. Important data suggesting a regulatory role of the MEM and Linker domains have been provided by targeted mutagenesis of *P. patens DEK1* (Demko et al., 2014; Johansen et al., 2016; Ako et al., 2017) and electrophysiology studies of plants with modified DEK1 accumulation in *A. thaliana* (Tran et al., 2017).

**Figure 2:**
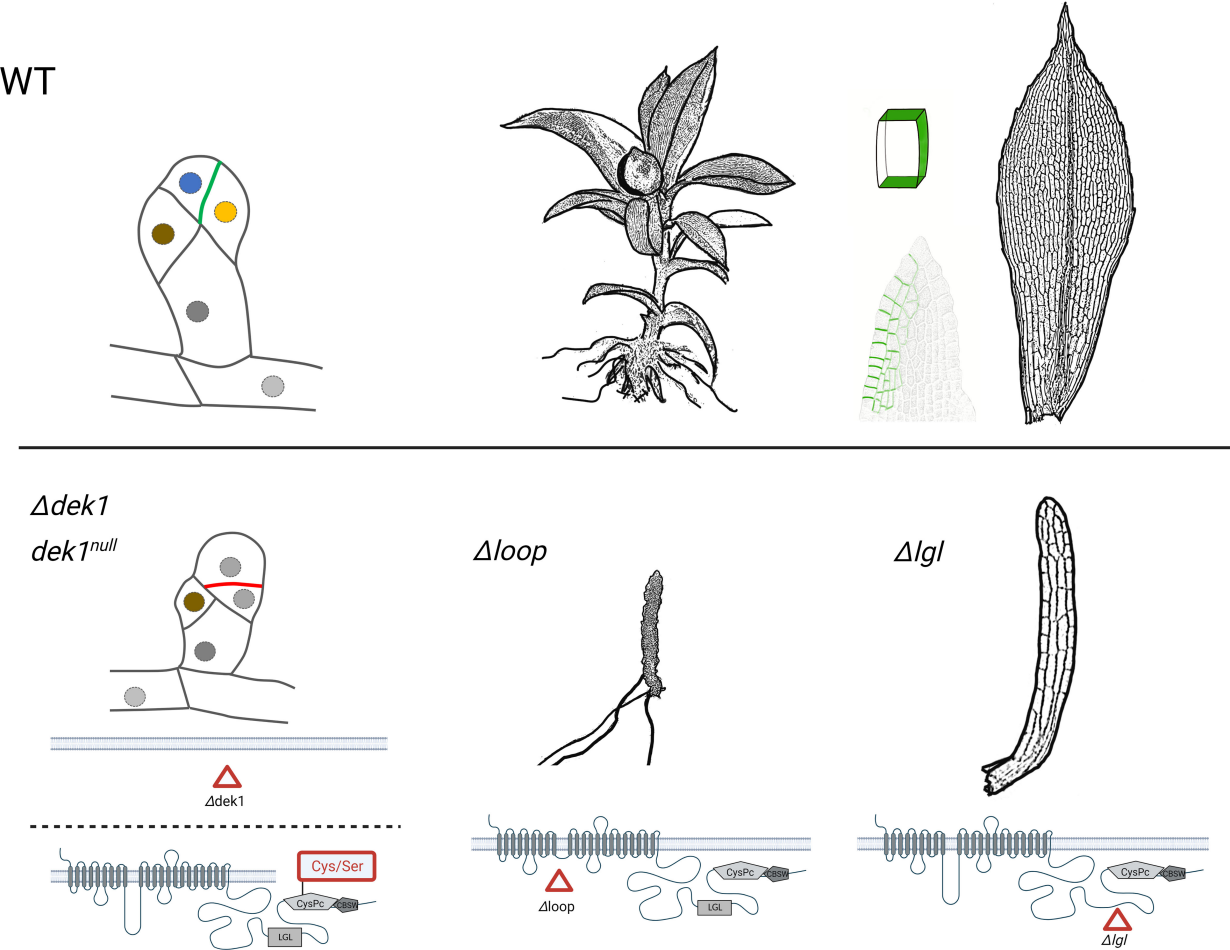
The schematic representation of *P. patens* bud development, the phenotype of gametophores resulting from various *DEK1* mutations. The wild type (WT) plants are compared with mutants after complete *DEK1* gene deletion (*Δdek1*), deactivation of DEK1 calpain (*dek1^null^*, substitution of Cys to Ser in the catalytic CysPc domain), deletion of *DEK1 Loop* (*Δloop*) and finally deletion of *DEK1 LGL* (*Δlgl*). In buds, the nuclei of individual cell types are marked in different colours. The polar distribution of PpDEK1_tdTomato at sites of cell-to-cell contacts is highlighted in green. Created with BioRender.com.

Deletion of *DEK1* gene in *P. patens* affects specific cell division in the bud (a juvenile meristematic structure that gives rise to leafy shoot gametophore). In wild type plants, a bud initial cell first divides asymmetrically to form an apical and a basal cell. The apical cell then undergoes a series of defined asymmetric cell divisions that form a bud apical stem cell and progenitor cells of surrounding tissues. Without DEK1, the bud apical cell fails to correctly orient division plane, which leads to early developmental arrest before the stem cell is established (Demko et al., 2014; Perroud et al., 2014) (**Figure 2**).

Deletion of the central Loop in PpDEK1 transmembrane domain abolished gametophore leaf (phyllid) formation, although naked stems with rooting structures (rhizoids) continued to grow (Demko et al., 2014) (**Figure 2**). This indicates that without the Loop, the calpain activity is sufficient to sustain gametophore apical stem cell divisions but fails to support phyllid development. Interestingly, the Loop segment is the least conserved part of DEK1 protein in land plants with 38% amino acid identity between *P. patens* and *A. thaliana* and 43% between *P. patens* and another bryophyte *Marchantia polymorpha*. Interspecies genetic complementation showed that reintroducing the DEK1 Loop from *M. polymorpha* into the *P. patens* mutant with deleted Loop completely rescued the mutant phenotype, however the Loop domains from *A. thaliana* and maize caused a new phenotype with altered leaf morphology (Johansen et al., 2016) indicating functional specialization of the Loop segment, or its interacting partners, in bryophytes and angiosperms.

Gametophore development and leaf morphology is specifically altered in *P. patens* mutants with deleted DEK1 LGL domain (**Figure 2**). Complete loss of the Linker segment caused severe phenotype with defective gametophore bud development. Deletion of the LGL domain however had no visible effect on early bud formation but affected the later development of gametophores that formed dramatically reduced narrow leaves. Deletion of the C-terminal part of the Linker, a 71 amino acid long segment between the LGL and calpain, severely disturbed early bud formation, and gametophores failed to develop (Johansen et al., 2016). It has been proposed that C-terminal segment of the Linker in *AtDEK1* contains a site for calpain autocatalytic cleavage, which mediates its activation (Johnson et al., 2008). On the other hand, when this segment was interrupted by in-frame insertion of the TdTomato fluorescent protein (475 aa long), normal gametophores were formed (Perroud et al., 2020) indicating its structural flexibility. Altogether, these data support a model where different developmental programs require fine-tuned DEK1 calpain activities controlled by mechanisms involving the transmembrane domain and Linker. The role of the DEK1 transmembrane domain in facilitating mechanosensitive Ca^2+^ transport has been proposed based on genetic and electrophysiology studies in *A. thaliana* (Tran et al., 2017). The authors proposed a scenario where mechanical cues within a cell wall are sensed by DEK1 transmembrane domain and transduced via locally elevated Ca^2+^, which would contribute to the calpain activation. Whether the DEK1 transmembrane domain forms a mechanosensitive ion channel remains unresolved and waits for the protein 3D structure determination.

One of the main unanswered questions about DEK1 is the identity of its calpain protease substrate(s). Based on genetic and biochemical analyses, several putative DEK1 calpain substrates have been proposed, all waiting for further experimental validation. Most recently, DEK1 activity has been associated with cellulose synthase (CESA) trafficking and mobility in the plasma membrane in *A. thaliana* epidermal cells (Novaković et al., 2023). The authors proposed that DEK1 might affect CESA indirectly via regulators of its intracellular dynamics, such as the cytoskeleton, the CSI/POM2 that facilitate interaction between cellulose synthases and cortical microtubules, the endo-1,4-beta-glucanase KORRIGAN involved in CESA regulation, and PATROL1 involved in the delivery of cellulose synthases to the plasma membrane. Whether the DEK1 calpain directly modulates/cleaves those proteins is currently unknown. Kim et al. (Kim et al., 2006) showed that a membrane-bound NAC transcription factor, the NTM1, is activated by proteolytic cleavage and mediates cytokinin signaling and cell division control in *A. thaliana*. Based on genetic studies of the NTM1 and detection of its truncated variants after diverse protease inhibitor treatments, the authors suggested that DEK1 calpain might be involved in NTM1 cleavage and hence its activation. The apparent similarity between the phenotypes of plants with overexpressed DEK1 (Tian et al., 2007b) and the ectopic expression of activated NTM1 further supports putative functional link between these two proteins. Using a similar approach, (Zhou et al., 2019) proposed that DEK1 calpain might be involved in the proteolytic cleavage of the BRI1 Associated Receptor Kinase 1 (BAK1). An additional intriguing target of the DEK1 calpain has been recently proposed in maize (Yi et al., 2011b; Wu et al., 2020). Based on genetic experiments, the authors concluded that DEK1 acts as a negative regulator of NOT1, the scaffolding protein of the CCR4-NOT complex. The CCR4-NOT complex is highly conserved in eukaryots and plays essential roles in regulating gene expression and cell fate determination (Denis and Chen, 2003; Collart and Timmers, 2004; Collart and Panasenko, 2012; Collart, 2016). While the *dek1* mutants fail to form aleurone cells in maize seeds, the *not1* mutants form multiple aleurone layers (Wu et al., 2020). The *DEK1* and *NOT1* are epistatic, where DEK1 is a negative regulator of NOT1, which is a negative regulator of aleurone cell fate (Yi et al., 2011b). Multiple transcriptional regulators have been predicted as potential DEK1 calpain substrates by (Demko et al., 2021) based on *P. patens* gene regulatory network analysis. The ultimate confirmation of the putative DEK1 targets as genuine *in vivo* substrates of the calpain requires additional experimental validation.

**Figure 3:**
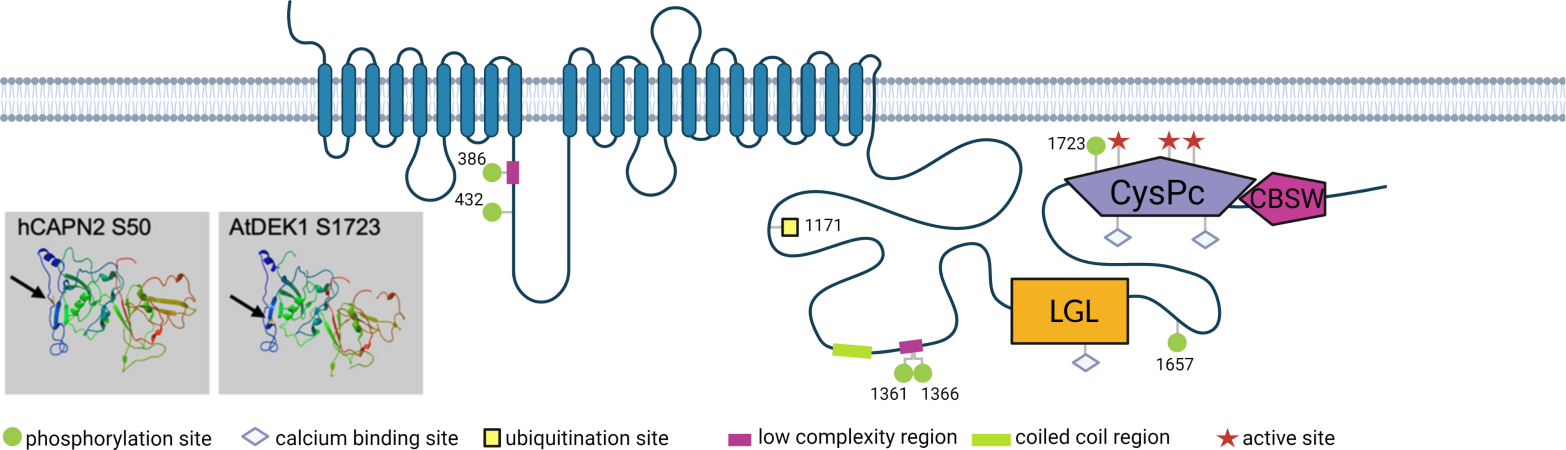
Schematic representation of AtDEK1 post-translational modifications. Numbers represent amino acid position of the modified site. Arrows point to phosphorylated serine in human CAPN2 (hCAPN2) and AtDEK1 in 3D structure models of the CysPc domains. The models were generated using the protein homology/analogy recognition engine Phyre^2^ (Kelley et al., 2015). Created with BioRender.com.

Post-translational modifications of DEK1 and their relevance for the protein regulation have not been experimentally investigated and represent yet another knowledge gap about this essential protein. In **Figure 3** we summarize detected sites of phosphorylation and ubiquitination of AtDEK1. Post-translational modifications have also been detected in rice and maize DEK1 proteins (**Table 1**). In *A. thaliana*, two phosphorylated serines have been detected in the DEK1 Loop, three in the Linker flanking the LGL domain, and one phospho-S site in the CysPc domain. In rice, one phospho-S site has been detected in the Loop and two in the Linker. One phospho-S site has been identified in the maize DEK1 Linker. Conserved amino acid positions of the phosphorylated serines in different plant species suggest the functional importance of this modification. Interestingly, the phosphorylated serine of AtDEK1 calpain (S1723) is in a similar position as previously analyzed S50 in human CAPN2 (**Figure 3**) (Sugiyama et al., 2008a). Phosphorylation of the S50 by ERK kinase has been implicated in the CAPN2 activation, even under the limited calcium availability (Glading et al., 2001). In the recent proteomic analysis of *A. thaliana* membrane proteins (Grubb et al., 2021a), a ubiquitination site has been detected in DEK1 at the position K1171 (**Figure 3**; **Table 1**). Ubiquitination of membrane proteins is essential in regulating their subcellular dynamics via vesicular trafficking and stability (Foot et al., 2017). Genetic and immunolocalization studies indicated that DEK1 is internalized from the plasma membrane and targeted to the endosomal pathway involving ESCRT protein SAL1 (Tian et al., 2007a). In *P. patens*, DEK1 shows the polar distribution, accumulating at sites of cell-to-cell contacts (Perroud et al., 2020) (**Figure 2**). In addition, the accumulation of PpDEK1 at the plasma membrane was shown to be developmentally regulated in gametophores and reproductive organs. The role of phosphorylation and ubiquitination in DEK1 subcellular dynamics is yet to be investigated.

**Table 1:** Post-translation modifications of DEK1.

Post-translational modification	Organism	Modified AA	Position	Peptide	References
Ph	*Arabidopsis thaliana*	S	386	SSSIDAGHTGCTNEANR	(Mergner et al., 2020) (Roitinger et al., 2015)
Ph		S	432	SEESGRPSLGLR	(Roitinger et al., 2015)
Ph		S	1361	AVGGDSVLEDSFAR	(Nühse et al., 2004)
Ph		S	1366	AVGGDSVLEDSFAR	(Roitinger et al., 2015)
Ph		S	1657	DFVMSVDSFAR	(Nühse et al., 2004; Roitinger et al., 2015)
Ph		S	1723	LQVVSEWMRPDSIVK	(Sugiyama et al., 2008b)
Ub		K	1171	DDVM**K**LR	(Grubb et al., 2021b)
Ph	*Oryza sativa ssp. japonica*	S	450	SNSCLSAVAVQDPETAVVSADR	(Nakagami et al., 2010; Wang et al., 2017b)
Ph		S	1377	AVGGDSALEDSFAR	(Wang et al., 2017b)
Ph		S	1668	DSVAIDIDSFAR	(Wang et al., 2017b)
Ph	*Zea mays*	S	1376	AVGGDSALEDSFAR	(Walley et al., 2013)

Ph, phosporylation; Ub, ubiqitination; S, Serine; K, Lysine. Underlined letters represent sites of modification in the peptide.

## 4 Four types of membrane-anchored calpains exist in eukaryotes

**Figure 4:**
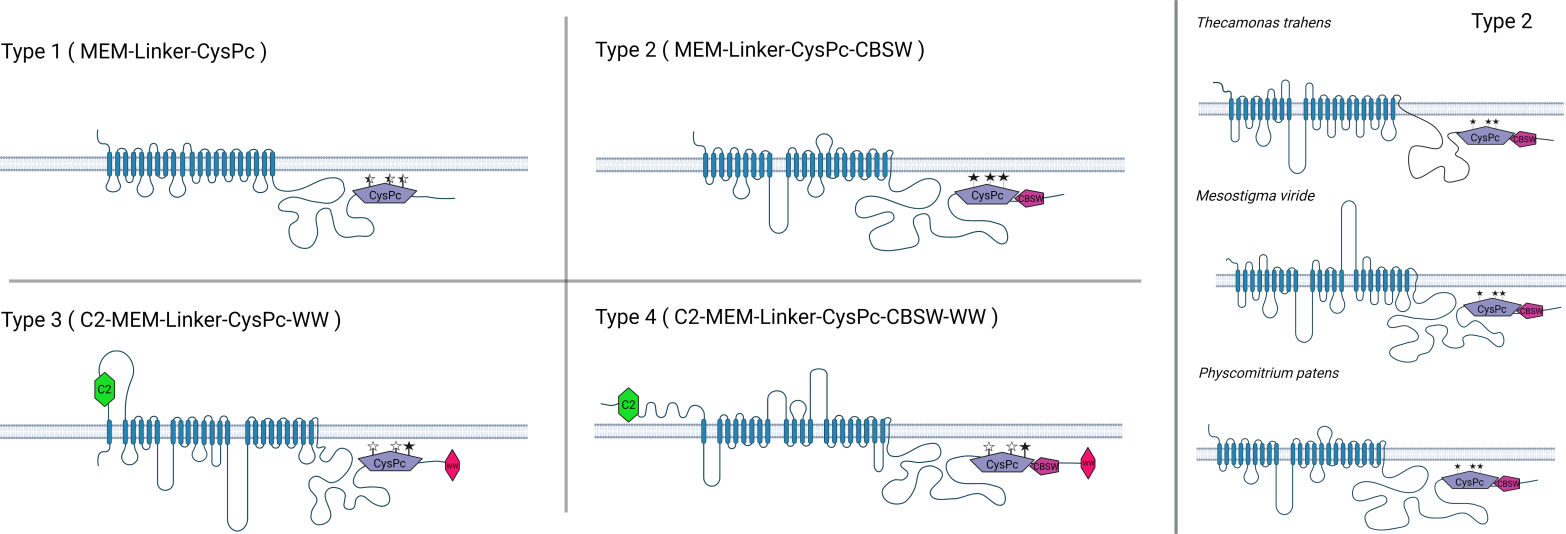
The members of Type 1 membrane-anchored calpains are primarily found in unicellular protozoans, including *Thecamonas trahens*, *Tetrahymena thermophila*, *Trichomonas vaginalis*. Type 2 members are represented by DEK1 protein found in charophyte algae and land plants as well as in protist calpains in *T. trahens*, *Stentor coeruleus* or *Cafeteria roenbergensis*. Type 3 and Type 4 membrane-anchored calpains are predominantly found in oomycetes such as *Phytophthora* sp., *Aphanomyces* sp., and *Albugo* sp. Created with BioRender.com.

Here, we screened available genome and proteome databases for calpain sequences containing a transmembrane domain. The vast majority of such calpains in the databases represent plant DEK1 proteins. Apart from plants, we identified 54 calpains with a well-defined protease core domain (CysPc) and a transmembrane domain (MEM). Based on predicted structural features, we sorted them into four major types (**Figure 4**; **Supplementary Table S1**). Type 1 members are composed of a transmembrane domain, a Linker and a calpain protease core domain CysPc (MEM-Linker-CysPc). Type 2 representatives consist of MEM, Linker, CysPc and a calpain-type beta-sandwich domain (MEM-Linker-CysPc-CBSW). Type 3 calpains contain an N-terminal C2 domain, MEM, Linker, CysPc and a WW domain (C2-MEM-Linker-CysPc-WW), and finally, type 4 members comprise a domain composition C2-MEM-Linker-CysPc-CBSW-WW (**Figure 4**). Within these well-defined domains, additional putative functional motives were predicted. These include a permease, a mechanosensitive channel, and a major facilitator superfamily (MFS) transporter motive within the MEM domain, or LGL domain within the Linker (for details see **Supplementary Table S1**). The LGL domain belongs to a concanavalin A-like lectin/glucanase domain superfamily with a typical β-sandwich fold structure. They contain Ca^2+^ binding site important for the LG fold (Hohenester, 2019). Proteins containing the Laminin G domain have been implicated in cell adhesion promoting activity, cell signaling, migration, membrane assembly and others (Aumailley, 2013; Paysan-Lafosse et al., 2023). Structural modeling of plant DEK1 LGL domain predicted eight antiparallel β-strands (Hohenester, 2019). Based on the bioinformatics analysis, LGL domains share 59% identity within land plants. However, very limited similarity to other proteins found in the NCBI database suggests their unique structure/function within the superfamily of concanavalin A-like lectin/glucanases (Johansen et al., 2016).

The function of C2 and WW domains in Type 3 and Type 4 calpains is unknown. Generally, the C2 domain is a calcium-binding domain involved in signal transduction and membrane trafficking (Farah & Sossin, 2012). The C2 domains have a wide range of lipid selectivity across major components of cell membranes, including phosphatidylserine and phosphatidylcholine. Furthermore, they are thought to play important roles in calcium-dependent phospholipid binding and membrane targeting processes (Davletov & Südhof, 1993). The 3D structure of the C2 domain is formed by an eight-stranded beta sandwich, which is wrapped around a conserved 4-stranded motif called the C2 key. The calcium binding sites are located in the recesses formed by the N- and C-terminal loops of the C2 key motif (Sutton et al., 1995).

The WW (also known as WWP domain or rsp5) is an approximately 40 amino acids long conserved domain with protein binding function often occurring as tamdems in a wide range of unrelated proteins (Andre & Springael, 1994; Bork & Sudol, 1994; Hofmann & Bucher, 1995; Sudol et al., 1995b). The name for this domain comes from two tryptophan residues 20 to 23 amino acids apart. The WW domain has a triple-stranded beta-sheet shape, binding either to specific proline motifs [AP]-P-P-[AP]-Y or to motifs containing phosphoserine-phosphothreonine (Chen & Sudol, 1995; Macias et al., 2002). The WW domain can be found in dystrophin, utrophin, YAP protein in vertebrates, mouse protein NEDD-4, yeast protein RSP5 and many others (Hofmann & Bucher, 1995; Sudol et al., 1995a).

The members of individual membrane-anchored calpain types vary in organization and length of their MEM and Linker domains (**Supplementary Figure S1**). As an example, for variability within the Type 2, the DEK1 protein from charophyte alga *Mesostigma viride* contains two long Loop segments in the MEM (153 aa and 225 aa long, respectively) in contrast to one central Loop (around 284 aa) in land plants DEK1 or the DEK1-like protein in the protist *Thecamonas trahens* (166aa long Loop) (**Figure 4**). The Linker in *T. trahens* is 336 aa long, while in *M. viride* 1293 aa and in land plants around 600 aa. Next, we analyzed the conservation of the CysPc active site residues Cys, His, and Asp in diverse membrane-anchored calpains (**Figure 4**; **Supplementary Table S1**). All three active site residues are conserved in Type 2 calpains. In Type 1, 3 and 4, the CysPc active site residues might be missing/substituted, indicating a putative non-proteolytic function of these calpains (for details, see **Supplementary Table S1**).

## Membrane-anchored calpains have an ancient evolutionary origin

Zhao et al. (Zhao et al., 2012) proposed that calpains with long transmembrane domains represent one of the four ancestral forms of calpain domain architectures in living organisms. In order to explore the phylogenetic distribution of different types of membrane-anchored calpains, we analyzed 232 calpain sequences, including 74 membrane-anchored members from 105 species representing the major taxonomic groups of eukaryotess. Using the CysPc domain sequence from the membrane-anchored members of the calpains and excluding N- and C-terminal truncated versions, we inferred a maximum likelihood (ML) phylogenetic tree.

**Figure 5:**
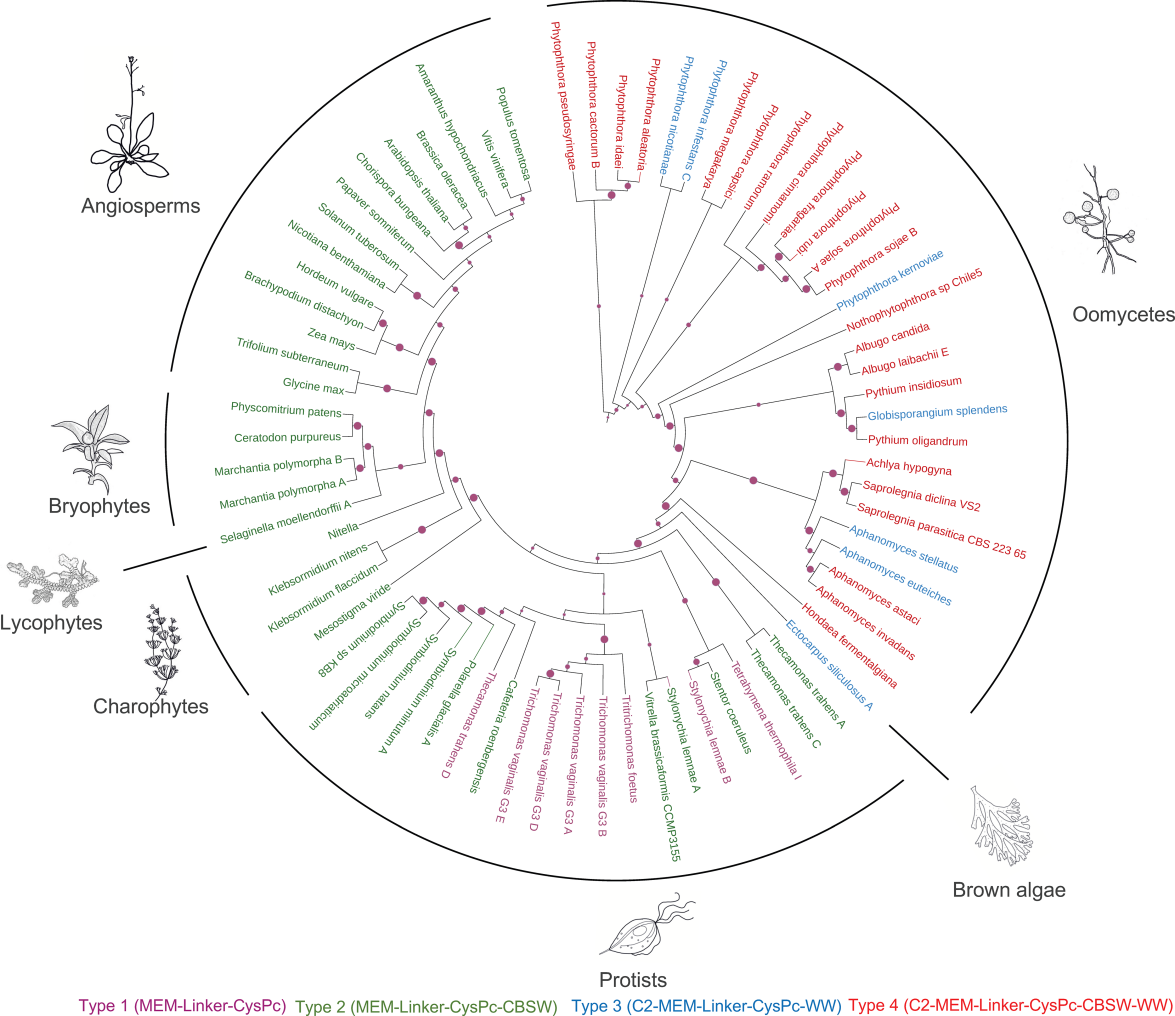
Maximum likelihood phylogenic tree of membrane-anchored calpains using a neighbour-joining method. The RPS (Reverse Position Specific) BLAST utility and the Conserved Domain Database (CDD) were used to identify and annotate conserved domains in the full-length calpain sequences. CysPc domains were retrieved by automatic annotation using the hmmsearch tool from the HMMER3 package (v. 3.1b1) and the PfamA-v.35 database. Multiple sequence alignment was performed using MAFFT v. 7 with the FFT-NS-I algorithm. The iTOl web server (Letunic & Bork, 2021) was used for the annotation of the resulting ML tree. Individual types of membrane-anchored calpains are highlighted by different colors: type 1 (MEM-Linker-CysPc) in purple; type 2 (MEM-Linker-CysPc-CBSW) in green; type 3 (C2-MEM-Linker-CysPc-WW) in blue; type 4 (C2-MEM-Linker-CysPc-CBSW-WW) in red.

Our phylogenetic analysis supports an ancient evolutionary origin of membrane-anchored calpains. Particularly, the members of Type 2 (MEM-Linker-CysPc-CBSW, in **Figure 5** highlighted in green) are present in taxons with the basal position in the evolutionary history of eukaryotic life such as the unicellular protist *Thecamonas trahens*. *T. trahens* belong to Apusomonadida, representing a sister group to the Opisthokonts, which include both fungi and animals (Cavalier-Smith & Chao, 2010). The Type 2 calpains are also distributed within the diverse SAR (Stramenopiles-Alveolates-Rhizaria) supergroup, including the freshwater ciliates *Stentor coeruleus* and *Stylonychia lemnae*, and bacterivorous marine flagellate *Cafeteria roenbergensis*, which has been positioned at the base of Stramenopiles (Heterokonts) evolution (Hackl et al., 2020). An additional member of the Type 2 calpains was found in the marine mixotrophic alga *Vitrella brassicaformis*. Interesitingly, Vitrella is a close relative to Apicomplexa (Mathur et al., 2018), a phylum represented by parasitic alveolate *Plasmodium falciparum* that contains only a single cytosolic calpain with an essential function in its cell cycle regulation (Russo et al., 2009; Soh et al., 2013), suggesting a loss of membrane-anchored calpains within this phylum of the SAR supergroup. The membrane-anchored calpains were also lost in the parasitic protists Trypanosoma and Leishmania (**Figure 5**). Their cytosolic calpains play an important role in cell morphogenesis and motility (Olego-Fernandez et al., 2009; Ennes-Vidal et al., 2019). Next, Type 2 calpains were found in closely related dinoflagellates *Polarella glacialis* and *Symbiodinium* sp. (**Figure 5**; **Supplementary Table S1**). The DEK1 protein is a sole membrane-anchored calpain in Streptophytes - harboring charophyte algae and land plants. A unique feature that distinguishes Streptophyte’s DEK1 from other Type 2 calpains is the presence of the LGL domain within the Linker segment (**Figure 3**; **Supplementary Table S1**), suggesting that acquisition of this domain was concomitant with the divergence of charophytes and chlorophytes that happened around one billion years ago (Leliaert et al., 2012). As previously reported (Zhao et al., 2012), no membrane-anchored calpains were found in chlorophytic algae.

Type 1 membrane-anchored calpains (MEM-Linker-CysPc, in **Figure 5** highlighted in purple) were found in diverse taxonomic groups, including protozoan flagellates *Thecamonas trahens* (Apusomonadida), *Tetrahymena thermophila* (Ciliophora), and *Trichomonas vaginalis* (Metamonada). Type 3 calpains (C2-MEM-Linker-CysPc-WW, in **Figure 5** highlighted in blue) were found in the brown alga *Ectocarpus siliculosus* and several oomycetes (**Figure 5**; **Supplementary Table S1**). Brown algae and oomycetes belong to Stramenopiles, the clade of SAR that diverged from plants, animals, and fungi more than billion years ago (Moustafa, 2009). Brown algae have unique position among Stramenopiles contributed to the evolution of multicellular bodies with differentiated tissues (Yoon et al., 2004). The members of Type 3 (C2-MEM-Linker-CysPc-WW, in **Figure 5** highlighted in blue) and Type 4 (C2-MEM-Linker-CysPc-CBSW-WW, in **Figure 5** highlighted in red) calpains were found predominantly in oomycetes including *Phytophthora* sp., *Plasmopara* sp., *Pythium* sp., *Aphanomyces* sp., *Saprolegnia* sp., *Achlya* sp., and *Albugo* sp. The results indicate early diversification of ancestral membrane-anchored calpains between SAR supergroup representatives and Streptophytes (charophyte algae and land plants) followed by early and later diversifications, as well as losses, within the SAR. Type 3 and 4 membrane-anchored calpains diversified early from Type 1 and 2, and represent lineage-specific forms.

## 5 Transient receptor potential channels and cytosolic calpains – an analogy to membrane-anchored calpains?

As mentioned above, membrane-anchored calpains have not been identified in vertebrates. Nevertheless, the function of animal cytosolic calpains is often tightly linked to cell membranes and transmembrane proteins. One intriguing example of transmembrane protein interaction with cytosolic calpains is TRPchannel vs. CAPN1 and CAPN2. The TRP channels belong to a superfamily of calcium-permeable nonselective cation channels anchored in cell membranes, usually by six membrane-spanning helices (Owsianik et al., 2006; Kaczmarek et al., 2012a). They assemble into homo- or heteromeric configurations, often with four units, thus 24 transmembrane spans forming a pore (Hellwig et al., 2005). The TRPs have not been found in Archaea and Bacteria, suggesting their eukaryotic origin (Himmel & Cox, 2020). There is no evidence of TRPs in land plants; however, TRP proteins have been found in chlorophyte algae (Wheeler & Brownlee, 2008; Arias-Darraz et al., 2015b), what is incidentally an opposite to situation with DEK1 calpains. Some TRPs are highly Ca^2+^ selective, while the vast majority of these proteins show cations’ nonselective attributes (Nilius & Owsianik, 2011). Widely studied mammalian TRP superfamily is divided into six subfamilies, including canonical (TRPC), vanilloid (TRPV), melastatin (TRPM), mucolipins (TRPML), ankyrin (TRPA), and polycystins (TRPP) (Koivisto et al., 2022). Apart from mammalian TRPs, the TRPN (no mechanoreceptor potential C, nompC) are found in invertebrates and fish, TRPY (yeast vacuolar conductance, YVC1) in yeasts and other fungi, and the TRP orthologs have also been found in protists (Palmer et al., 2001; Nilius & Owsianik, 2011; Ahmed et al., 2022; Hellmich & Delemotte, 2022). The TRPs are polymodal channels acting as the integral membrane sensors of various environmental stimuli participating in mechanosensation, thermosensation, taste and light perception in animals and humans. The TRP channels are highly permeable to Ca^2+^ ions and contribute to membrane potential, intracellular Ca^2+^ fluxes and cell signaling (Arias-Darraz et al., 2015a; Khalil et al., 2018; Samanta et al., 2018). In mice podocytes, the TRPC6 binds to cytosolic calpains CAPN1 and CAPN2 and, independent of its channel activity, regulates cytoskeleton, cell adhesion and motility (Farmer et al., 2019). Here, the TRPC6 acts as a scaffolding protein that localizes calpain near the membrane in order for it to cleave adhesive and cytoskeletal proteins. In another example, the TRPV1 was shown to activate calpain during nociception (Wang et al., 2017a; Farmer et al., 2019). On the other hand, mammalian CAPN1 and CAPN2 have been shown to cleave and activate TRPC5 acting in neuronal growth (Kaczmarek et al., 2012b). Thus, hypothetically, the functional association of TRP channels and cytosolic calpains could provide a hint at intramolecular functions within membrane-anchored calpains.

## 6 Membrane-anchored calpains as targets for future functional studies

Calpain proteases play essential functions in animals and plants. However, being essential implies a potential danger when such proteins are misregulated. Compromised calpain functions have been associated with severe pathologies in humans. Loss-of-function mutations of plant’s sole membrane-anchored calpain DEK1 are embryo lethal and its misregulation leads to dramatic developmental alterations. To understand the molecular function of calpains, extensive genetic and structural studies have been conducted over the past decades, mostly focused on classical animal calpains. These works brought invaluable data about the pathways and mechanisms of calpain activation and substrate processing, which allowed to design tools interfering with specific calpain functions. Apart from biomedical applications, the research of calpain’s mode of action continues to uncover fundamental biological and cellular processes. Although membrane-anchored calpains have not been detected in yeasts, worms, insects, or vertebrates, they are distributed in organisms with utmost importance for ecosystems and humankind, such as plants and beneficial and pathogenic microorganisms.

Land plants represent the only organisms where membrane-anchored calpain (DEK1) has been studied and shown to play essential role throughout ontogenesis. Although the indispensable function of DEK1, many questions need to be addressed in order to understand how this protein works at the molecular level and in the context of other regulatory pathways. These include solving its 3D structure and identifying the cellular substrates of its calpain. Recent progress in the development of tools for gene editing in plants opens doors for experiments investigating the regulation of DEK1 via posttranslational modifications, including phosphorylation, calcium binding, and ubiquitination.

Beyond plants, membrane-anchored calpains with completely unknown functions are present in diverse microorganisms occupying various habitats, including ice blocks in the Arctic and Antarctic regions, freshwaters, rhizosphere, and seas. Most membrane-anchored calpain-containing oomycetes are parasites causing significant economic losses in crops as well as animal and human diseases (Derevnina et al., 2016a; Chaimon et al., 2019). Uncovering any protein’s developmental and molecular functions require a suitable model organism and apropriate genetic and cell biology tools. Below, we discuss selected species whose membrane-anchored calpains might be addressed for future functional studies.

*Tetrahymena thermophila* is a unicellular ciliate and established model organism for experimental studies focusing on genome organization, cell cycle regulation, reproduction and cytoskeleton dynamics (Eisen et al., 2006; Ruehle et al., 2016). *T. thermophila* contains both cytosolic calpains and a Type 1 membrane-anchored calpain (MEM-Linker-CysPc) with conserved active site residues Cys, His, Asp in the catalytic domain (**Supplementary Table S1**). The availability of forward and reverse genetic tools, including homologous recombination-mediated DNA mutagenesis and RNA interference (Chalker, 2012) makes *T. thermophila* a suitable model system for discovery of yet unknown function(s) and substrates of membrane-anchored calpain in a single-celled organism.

*Stentor coeruleus* is a giant unicellular ciliate and experimental model for cellular regeneration, cell polarity formation, mechanosensing and cellular habituation (Kirschner et al., 2000; Mine et al., 2008; Marshall, 2011; Rajan et al., 2023). Stentor contains a single Type 2 membrane-anchored calpain (MEM-Linker-CysPc-CBSW) with conserved active site residues alongside with cytosolic calpains (**Supplementary Table S1**). RNA interference through cyanobacteria feeding has been recently used for gene downregulation in Stentor (Wei et al., 2021). Immunolabeling methods and proteomics data are also available (Maloney et al., 2005). Interestingly, a polar (posterior) distribution of calpains has been detected in Stentor (Lin et al., 2022). Whether the membrane-anchored calpain is involved in cell polarity establishment and single-celled body patterning using this uniquely large ciliate remains to be uncovered.

*Ectocarpus siliculosus* is a marine brown alga that has been used as a model organism for the evolution of multicellularity (Cock & Collén, 2015; Cock et al., 2015). It contains a Type 3-like membrane-anchored calpain (C2-MEM-Linker-CysPc-WW) and several cytosolic calpains (**Supplementary Table S1**). In brown algae, the multicellularity and body patterning mechanisms evolved independently from animals, plants, fungi, and red algae (Cock & Collén, 2015). As discussed above, the differentiation of tissue layers and body patterning in plants depends on DEK1 activity. Therefore, it would be very exciting to investigate the role of membrane-anchored calpain in the body patterning of brown algae. Although forward genetics tools have been successfully applied (Coelho et al., 2020), reverse genetics methods such as RNA interference and CRISPR/Cas are still being developed in Ectocarpus (Badis et al., 2021).

Several oomycete species contain Type 3 (C2-MEM-Linker-CysPc-WW) and Type 4 (C2-MEM-Linker-CysPc-CBSW-WW) membrane-anchored calpains (**Figure 5**, **Supplementary Table S1**). Most of them are parasitic organisms causing severe diseases in crop plants and animals. Hazardous crop pathogens include *Phytophthora* sp. and *Plasmopara* sp. (Derevnina et al., 2016b). Both plants and animals are being infected by Pythium and Aphanomyces species. *P. oligandrum* also attacs other oomycetes and fungi (Sankaranarayanan & Amaresan, 2020). *P. insidiosum* cause destructive tissue lesions, a severe disease called *pythiosis*, in animals and humans(Sathapatayavongs, 2012). *A. stellatus* and *A. euteiches* with Type 3 membrane-anchored calpains are plant pathogens causing root rot (Becking et al., 2022). *A. invadans* and *A. astaci* with Type 4 calpains infect fish and crayfish, causing epizootic ulcerative syndrome and crayfish plague, respectively (Iberahim et al., 2018; Chong, 2022). The application of tools for genetic transformation, gene silencing and targeted mutagenesis in oomycetes is still challenging. Nevertheless, electroporation, biolistics, PEG-mediated protoplast transfection, and Agrobacterium-mediated transformation methods have been successfully used in selected oomycetes. Pioneering studies using RNA interference and CRISPR/Cas in Phytophthora species and *Aphanomyces invadans* showed the potential to modulate their development and virulence using reverse genetics tools [revived in (Ghimire et al., 2022)].

## 7 Concluding remarks

The calpain superfamily is comprised of structurally diverse members. They differ in domain composition, expression pattern, subcellular distribution, and proteolytic activity. The research of animal calpains uncovered critically important functions of calpains during normal development as well as their involvement in diseases and pathologies. Although their functions are often associated with biological membranes, the animal calpains are cytosolic. The first membrane-anchored calpain DEK1 has been discovered in maize and later shown to play essential roles in the development of land plants. As we present here, apart from plants, the genes encoding similar types of membrane-anchored calpains have been found in a number of diverse organisms, including unicellular protists, brown algae, and oomycetes, the species with significant biomedical, agricultural, and ecological impact. Their function is still being determined. Given the conserved essential roles of DEK1 in the evolutionary distant groups of plants, one could expect that DEK1-like proteins also play important roles in Trichomonas, Stentor, Ectocarpus, Aphanomyces, Phytophthora. Membrane-anchored calpains represent a challenge for structural biology. Apart from partial homology to diverse major facilitator superfamily channels described in DEK1, the calpain’s multi-spanning transmembrane domain show no homology to any known protein and likely represents a unique structure. Whether it is a mechanosensitive channel, a positional cues-mediating transceptor, or “simply” an anchor for multiprotein complexes is yet to be discovered. Advanced approaches for gene editing, protease substrates identification, and protein-protein interactions could shed light not only on the molecular function and regulation of diverse membrane-anchored calpains but expand our knowledge about calpain’s regulation and cellular functions beyond plants.

## Data availability statement

The original contributions presented in the study are included in the article/**Supplementary Material**. Further inquiries can be directed to the corresponding author.

## Author contributions

MŠ: Investigation, Visualization, Writing – original draft. AS: Data curation, Formal analysis, Investigation, Methodology, Writing – original draft. WJ: Formal analysis, Investigation, Methodology, Software, Writing – original draft. JŠ: Data curation, Formal analysis, Writing – review & editing. SV: Data curation, Formal analysis, Investigation, Writing – original draft. BB: Data curation, Formal analysis, Methodology, Visualization, Writing – original draft. JJ: Supervision, Writing – review & editing. VD: Conceptualization, Funding acquisition, Supervision, Visualization, Writing – original draft, Writing – review & editing.
