# Supplementary material for: Membrane-anchored calpains – hidden regulators of growth and development beyond plants?
Source: Front Plant Sci. 2023 Dec 19;14:1289785. doi: 10.3389/fpls.2023.1289785 (PMC10762896; doi:10.3389/fpls.2023.1289785)

Fig. S1. A list of SMART-predicted domain composition of all membrane-anchored calpains retrieved for this work.

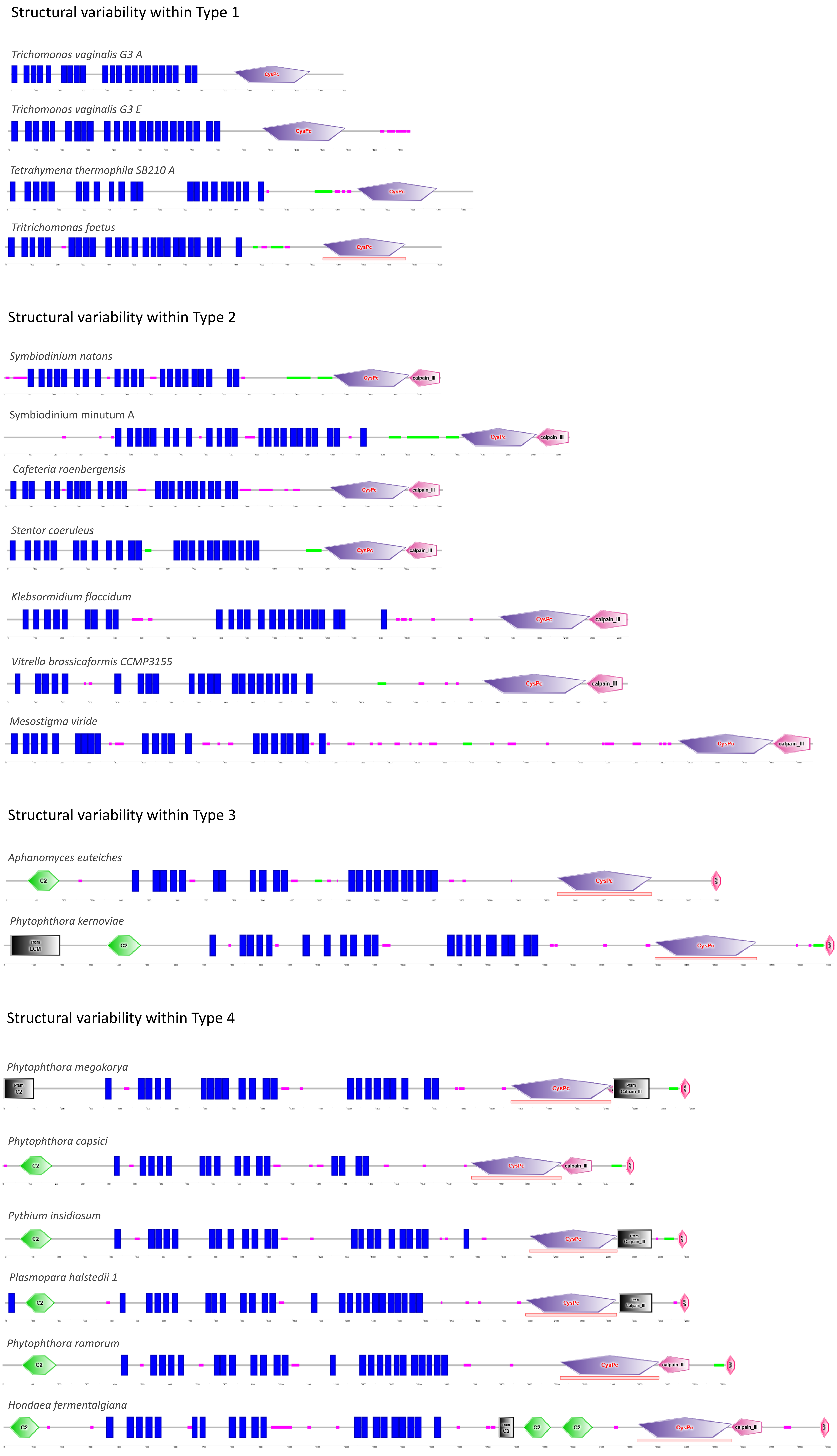

Supplement: Supplementary file 1 [file DataSheet_1.pdf]
